# A Novel Effector FoUpe9 Enhances the Virulence of *Fusarium oxysporum* f. sp. *cubense* Tropical Race 4 by Inhibiting Plant Immunity

**DOI:** 10.3390/jof11040308

**Published:** 2025-04-13

**Authors:** Zheng Cong, Yini Ma, Lisha Zeng, Yaoyao Wu, Yaojun Chen, Ludan Liang, Jie Zhu, Huaping Li, Yanfang Nie, Yunfeng Li

**Affiliations:** 1 Guangdong Province Key Laboratory of Microbial Signals and Disease Control, College of Plant Protection, South China Agricultural University, Guangzhou 510642, China; ddcongzheng@icloud.com (Z.C.); mayinnnni@163.com (Y.M.); wuyaoy2000@163.com (Y.W.); ludanliang0128@163.com (L.L.); hnzhujie2023@163.com (J.Z.); huaping@scau.edu.cn (H.L.); 2 Dongguan Agricultural Research Centre, Dongguan 523106, China; kitygirl11@126.com; 3 College of Materials and Energy, South China Agricultural University, Guangzhou 510642, China; c1793790102@163.com

**Keywords:** banana Fusarium wilt, *Fusarium oxysporum* f. sp. *cubense*, effector, virulence, immune responses

## Abstract

Fusarium wilt caused by *Fusarium oxysporum* f. sp. *cubense* tropical race 4 (Foc TR4) is the most destructive disease of the banana. Effectors play a crucial role in Foc TR4–banana interaction; however, only a few effectors have been functionally characterized. Our previous secretome studies on Foc TR4 highlighted an uncharacterized protein without any conserved domains (named FoUpe9), which was predicted to be a candidate effector. Herein, bioinformatics analysis showed that FoUpe9 was highly conserved among *Fusarium* species. *FoUpe9* was highly induced during the early infection stages in the banana. A yeast signal sequence trap assay showed that FoUpe9 is a secretory protein. FoUpe9 could inhibit cell death and ROS accumulation triggered by BAX through the *Agrobacterium*-mediated *Nicotiana benthamiana* expression system. Subcellular location showed that FoUpe9 was located in the nucleus and cytoplasm of *N. benthamiana* cells. Deletion of the *FoUpe9* gene did not affect mycelial growth, conidiation, sensitivity to cell-wall integrity, or osmotic and oxidative stress, but significantly attenuated fungal virulence. *FoUpe9* deletion diminished fungal colonization and induced ROS production and expression of SA-related defense genes in banana plants. These results suggest that FoUpe9 enhances Foc TR4 virulence by inhibiting host immune responses and provide new insights into the functions of the uncharacterized proteins, further enhancing our understanding of effector-mediated Foc TR4 pathogenesis.

## 1. Introduction

The banana (*Musa* spp.) is one of the world’s most important fruits [1]. Banana Fusarium wilt (also known as Panama disease), caused by *F*. *oxysporum* f. sp. *cubense* (Foc), is one of the most destructive diseases and causes tremendous economic loss in the global banana industry [2,3]. Foc invades banana roots and causes wilt disease through colonization in xylem vessels [4]. The classic symptoms of banana Fusarium wilt include yellowing of the lower leaves, progressive wilting, and even plant death [5]. Foc has been classified into three physiological races (race 1, race 2, and race 4) based on their host scope [6]. Foc4 has been further classified into tropical race 4 (Foc TR4) and subtropical race 4 (Foc STR4) according to their geographical location range [7,8]. Foc TR4 is considered the most destructive race and it is particularly serious in China because it is the biggest producer of Cavendish bananas (AAA) in the world [9].

In nature environments, plants defend against various pathogens through physical barriers and endogenous immune systems [10,11]. Plants’ transmembrane pattern recognition receptors (PRRs) can effectively respond to the pathogen-associated molecular patterns (PAMPs) [12]. However, pathogens can enhance their adaptability by secreting effectors into plant cells during the co-evolutionary process with their hosts, to suppress the plant’s immune responses [13,14]. Plants can also use receptors encoded by resistance genes to recognize these specific effectors and trigger stronger immune responses called effector-triggered immunity (ETI), resulting in disease resistance and hypersensitive reaction at the infection site [15,16].

Foc TR4 is thought to secrete a repertoire of effectors to modulate host physiological processes during infection, from which only about 10 candidate effectors have been identified [17,18,19]. For example, SIX8 protein, secreted in xylem 8 protein, was found to be an essential virulence effector of Foc TR4, which had only been detected in Foc TR4 and allowed Foc TR4 to be distinguished from Foc1 [20]. FoCupin1, a cupin type-1 domain-containing protein, could suppress BAX-induced cell death in *N. benthamiana* and is an essential virulence effector of Foc TR4 [21]. FocRnt2, a ribonuclease protein belonging to the T2 family, inhibits cell death and ROS accumulation in *N. benthamiana* induced by BAX and contributes to the virulence of Foc TR4 [22]. However, many effectors from phytopathogenic fungi, including Foc TR4, rarely possess conserved motifs and domains, and the biological functions of these effectors are poorly understood [17,23,24].

In our previous work, we performed a shotgun secretome analysis of Foc TR4, and 70 candidate effectors were predicted by bioinformatics tools [17]. Herein, we characterized a candidate effector, an uncharacterized protein (named FoUpe9) from the Foc TR4 secretome, which was highly induced during the early stages of banana infection by Foc TR4. FoUpe9 is a classically secreted protein without any known domain, which may represent a novel candidate effector. Therefore, the objective of this study was to explore the functions of FoUpe9 in Foc TR4. In this study, we found that FoUpe9 is essential for fungal virulence and suppresses plant immunity. These findings provide critical insights into the detailed molecular mechanism of FoUpe9 and improve our understanding of Foc TR4 pathogenicity.

## 2. Materials and Methods

### 2.1. Fungal Strains, Plant Materials, and Growth Conditions

The wild-type strain DZ1 and all the mutants of Foc TR4 generated in this study were routinely cultured on PDA plates at 28 °C. To test sensitivity against different stresses, the DZ1 strain and mutant strains were cultured on regular PDA plates with 1 mol/L NaCl, 1 mol/L sorbitol, 0.02% *w*/*v* SDS, 100 μg/mL CR, 50 μg/mL CFW, or 30 mM H_2_O_2_ at 25 °C for 7 days. Conidiation was assayed with 3-day-old liquid Czapek Dox cultures [21]. The banana cultivar Brazilian (AAA group, Cavendish) was used in this study, which is susceptible to Foc TR4. Banana seedlings at the fourth-leaf stage were used for all experiments. Five-week-old *N. benthamiana* was used for all transient assays as described previously [25].

### 2.2. Bioinformatic Analysis

The sequence of FoUpe9 protein (gene name: *FOIG_05467*) and its homologs from different phytopathogens were retrieved from the NCBI GenBank database and aligned using ClustalX 2.1. Phylogenetic trees were constructed using MEGA 11.0 with the neighbor-joining method and 1000 bootstraps. The Simple Modular Architecture Research Tool (SMART) was used to search conserved domains of FoUpe9 protein. Classically secreted protein features were analyzed by SignalP 6.0, TMHMM 2.0, Big-PI Fungal Predictor server (https://mendel.imp.ac.at/gpi/fungi_server.html, last accessed: 18 February 2025), and WoLF PSORT (https://wolfpsort.hgc.jp/, last accessed: 18 February 2025) as described [17]. The FoUpe9 sequence was submitted to EffectorP 3.0 for effector prediction.

### 2.3. Yeast Signal Sequence Trap System

The proteins from 8 h and 24 h collection were mixed in a 1:1 (*w*/*w* total protein). Secretion assay was performed as described previously [22]. In brief, the SP sequence of FoUpe9 was cloned into the pSUC2 vector, which contained a truncated invertase gene but lacked a signal peptide. The resulting vectors were separately transformed into the YTK12 yeast strain. The YTK12 strain carrying pSUC2:Avrb was used as a positive control, while the YTK12 strain and the YTK12 stain carrying empty vector pSUC2 were used as negative controls. All transformed yeast strains were cultured on YPDA medium, CMD-W medium without tryptophan, and YPRAA medium containing raffinose as the only carbon source, respectively. The reduction of TTC to the insoluble red-colored triphenyl formazan was used to assess invertase activity [26]. All the experiments were repeated three times.

### 2.4. Real-Time Quantitative PCR (RT-qPCR) Assays

Total RNA was extracted from Foc TR4, banana, and tobacco using the Fungal RNA kit (Omega, Knoxville, TN, USA) and Plant RNA Kit (Omega, Knoxville, USA) according to the manufacturer’s instructions, respectively. RT-qPCR was performed on a CFX Coxnnect™ Real-Time System (Bio-Rad, Hercules, CA, USA) with the SYBR Premix Ex Taq Kit (TaKaRa, Beijing, China) according to the manufacturer’s instructions. The housekeeping genes *FoEF1α*, *MaActin*, and *NbEF1α* were used as internal references of Foc TR4, banana, and *N. benthamiana*, respectively. Relative expression levels were analyzed as previously described [27]. All primers used in RT-qPCR were listed in Table S1. All experiments were repeated three times.

### 2.5. Agroinfiltration Assays

FoUpe9 with or without the signal peptide (named SPFoUpe9 or NSPFoUpe9) was separately inserted into the pBI21 plasmid. The recombinant constructs were transformed into *A. tumefaciens* GV3101 through heat-shock transformation. Subsequently, 4-week-old *N. benthamiana* leaves were infiltrated as described [28]. The pBI121 plasmid carrying Bcl2-associated X protein (BAX) and translationally controlled tumor protein (TCTP) served as positive and negative controls. Total proteins were extracted using a Plant Protein Extraction Kit (Beyotime Institute of Biotechnology, Shanghai, China) from agroinfiltrated *N. benthamiana* leaves 48 h after infiltration. Transient protein expression in *N. benthamiana* was assessed using mouse antibodies against actin (Sigma-Aldrich, St. Louis, MO, USA) or rabbit antibodies against HA (CST, Danvers, MA, USA). All *N. benthamiana* leaves were photographed 4 days after infiltration, and the experiment was repeated three times.

### 2.6. Deletion and Complementation of FoUpe9

The *FoUpe9* gene deletion strains (∆*FoUpe9*) and complemented strains (∆*FoUpe9*-com) were constructed using the PEG-mediated transformation method as described [21]. The deletion mutants were confirmed by PCR, RT-qPCR, and southern blot analysis. All complemented strains were verified by PCR and RT-qPCR analysis. All primers used in this assay are listed in Supplementary Table S1.

### 2.7. Stress Sensitivity Assays

To test fungal sensitivity to different stress, Foc TR4 and the mutant strains were cultured on PDA plates containing final concentrations of 1 mol/L NaCl, 1 mol/L sorbitol, 0.02% *w*/*v* SDS, 100 μg/mL congo red (CR), 50 μg/mL calcofluor white (CFW), or 30 mM H_2_O_2_, respectively. All plates were cultured at 28 °C for 5 days in the dark.

### 2.8. Pathogenicity Tests

Virulence assays were performed as described previously [21]. Briefly, the roots of banana seedlings at the fourth-leaf stage were soaked in fungal conidial suspension (1 × 10^5^ mL) for 30 min. The banana seedlings were then transplanted into nutrient soil and cultivated in a greenhouse at 26 °C. The disease symptoms were measured 21 days post-inoculation. To evaluate the fungal biomass, the inoculated banana roots were collected at 24, 48, 72 h and 5, 10, 15 days, respectively. DNA-based qPCR was performed and the relative fungal biomass was assessed as described previously [29].

### 2.9. Subcellular Localization

To perform transient expression assays, SPFoUpe9 or NSPFoUpe9 protein was fused separately to EGFP at its C-terminus in the pBI121 vector. Subsequently, the recombinant constructs were individually transformed into the H2B-mCherry transgenic *N. benthamiana* leaves using an *Agrobacterium*-mediated transformation system. H2B-mCherry protein was used as a nuclear marker. The inoculated *N. benthamiana* leaves were collected after 2  days and observed with a Leica TCS SP8 laser scanning confocal microscope (Germany). Images were acquired and processed using Leica Application Suite X software, version 4.12.0 (Leica Microsystems GmbH, Mannheim, BW, Germany).

### 2.10. DAB Staining and H_2_O_2_ Measurements

*N. benthamiana* leaves and banana roots were sampled after agroinfiltration or fungal inoculation. A staining solution of 3,3′-diaminobenzidine (DAB) was used to visualize ROS accumulation in plant tissues. *N. benthamiana* leaves were decolored with ethanol, acetic acid, and glycerol mixture liquid in a boiling water bath until chlorophyll disappeared. Quantitative measurement of H_2_O_2_ was detected by the titanium tetrachloride precipitation method as described [30]. The experiments were repeated three times.

### 2.11. Statistical Analysis

Data analysis and processing were performed using GraphPad Prism 9 software. To ensure that the differences were statistically significant, experimental data were measured in triplicate and expressed as mean ± SE. Duncan’s multiple-range test was used to analyze the significance of differences between groups at a 5% probability level.

## 3. Results

### 3.1. FoUpe9 Is Highly Conserved in Fusarium Genus

**Figure 1:**
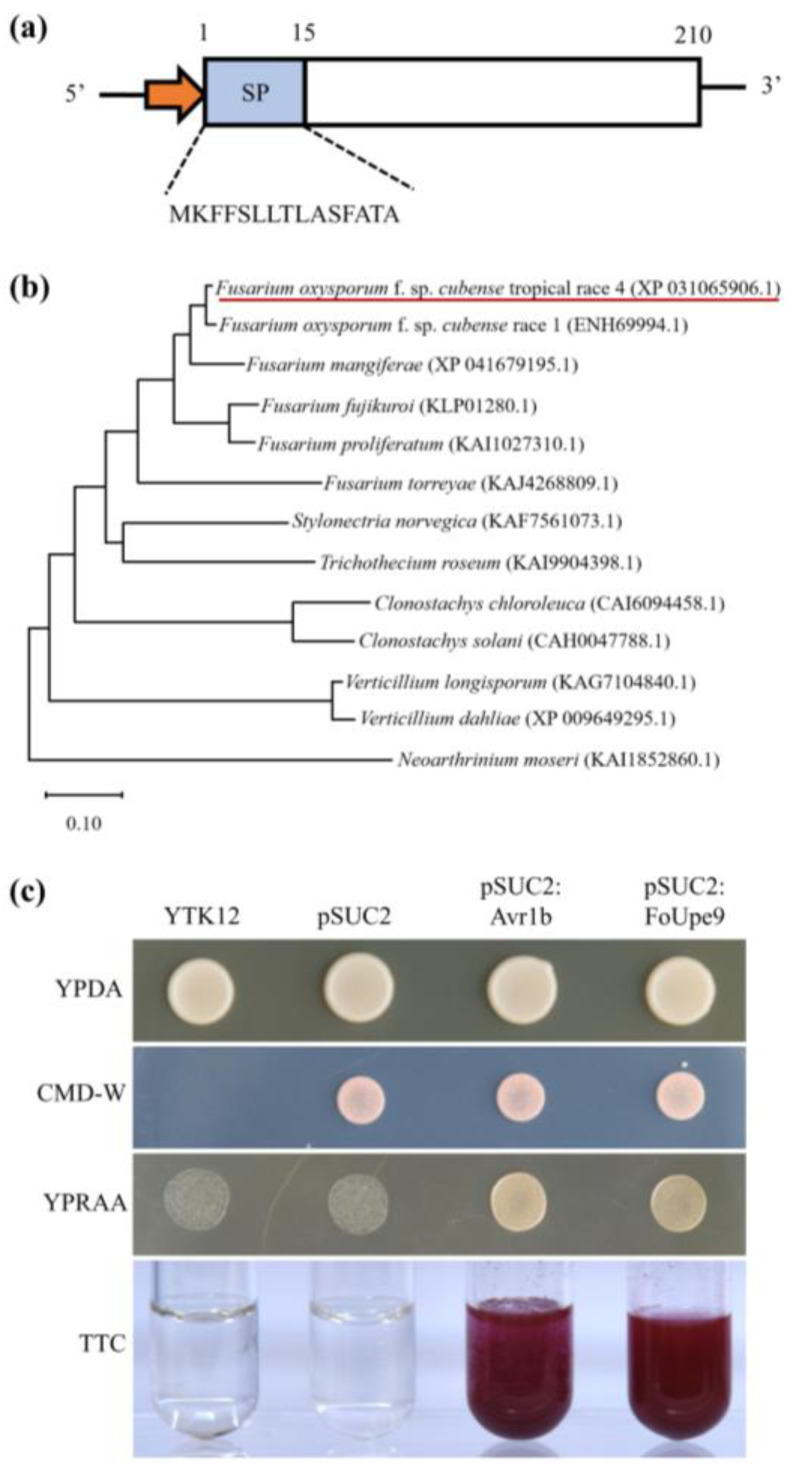
FoUpe9 protein contains a signal peptide with secretion function. (**a**) Structure diagram of FoUpe9 protein. The signal peptide (SP) was predicted by SignalP 5.0. (**b**) Phylogenetic analysis of FoUpe9 protein and its orthologous proteins from thirteen phytopathogenic fungi. The red underline indicates the FoUpe9 protein. (**c**) Secretion functional validation of the SP of FoUpe9. The yeast YTK12 strain carrying the pSUC2:FoUpe9 vector (FoUpe9 SP sequence fused in the pSUC2 vector) was able to grow on the CMD-W and YPRAA plates and could induce a red color reaction. The YTK12 strain carrying pSUC2:Avr1b served as a positive control, while the YTK12 strain and YTK12 strain carrying the pSUC2 empty vector served as negative controls. The change in color of TTC was used to confirm the enzymatic activity.

FoUpe9 is encoded by the *FOIG_05467* gene and contains 210 amino acids without any known functional domain. FoUpe9 has a signal peptide of 15 amino acid residues at the N-terminus, lacks the transmembrane domain or GPI-anchor site, and exhibits the characteristics of a classically secreted protein (Figure 1a). Phylogenetic tree analysis indicates that homologous proteins of FoUpe9 are widely present in phytopathogenic fungi (Figure 1b). Sequence alignment analysis using BLASTp (https://blast.ncbi.nlm.nih.gov/Blast.cgi, last accessed: 18 February 2025) against the NCBI database shows that FoUpe9 shares significant sequence similarity with several uncharacterized proteins from *Fusarium* species (Figure S1), such as uncharacterized protein (ENH69994.1; similarity: 97.62%) from *F. oxysporum* f. sp. *cubense* race 1, uncharacterized protein (KAG7418753.1; similarity: 93.81%) from *F. oxysporum* f. sp. *rapae*, and uncharacterized protein (XP041679195.1; similarity: 91.90%) from *F. mangiferae*.

### 3.2. FoUpe9 Contains a Functional Signal Peptide

To assess the secretion activity of FoUpe9 protein, its signal peptide (amino acids 1–15) was cloned into the pSUC2 vector, which was then transformed into the invertase-deficient yeast strain YTK12. All yeast strains were able to grow on YPDA plates, and the strains containing the pSUC2 vector could grow on CMD-W plates (Figure 1c). However, only the strains containing the pSUC2 vector harboring a signal peptide fragment with secretion function could grow on YPRAA plates and reduce 2,3,5-triphenyl tetrazolium chloride (TTC) to form red triphenyl formazan (Figure 1c). The yeast strain carrying pSUC2:Avr1b served as a positive control, while YTK12 and YTK12 strains carrying the empty pSUC2 vector served as negative controls. These results reveal that FoUpe9 harbors a functional signal peptide.

### 3.3. FoUpe9 Is Highly Expressed During the Early Infection Stage

**Figure 2:**
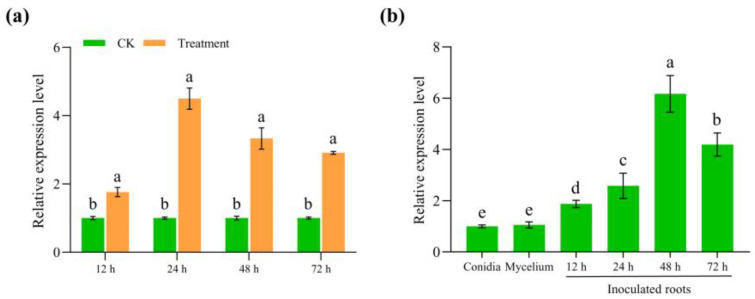
RT-qPCR analysis of *FoUpe9* expression. (**a**) Expression of the *FoUpe9* gene in Foc TR4 cultured on NCM medium (CK) and NCM medium supplemented with banana plant extracts (Treatment). (**b**) Expression of the *FoUpe9* gene in mycelium, conidia, and infection stages. *FoEF1α* gene was used as the internal reference. Values are the means based on three independent experiments, and bars indicate standard deviations. Different letters indicate statistical significance (*p* < 0.05) using Duncan’s new multiple-range method.

To determine the expression levels of *FoUpe9* in banana–Foc TR4 interaction, RT-qPCR analysis was performed by using the in vitro method and in planta method, respectively [22]. The expression levels of *FoUpe9* in Foc TR4 were significantly induced and peaked at 48 h by culturing fungal conidia in NCM medium supplemented with plant extracts to mimic banana–Foc TR4 interaction in vitro (Figure 2a). Furthermore, *FoUpe9* was also significantly up-regulated during the early stages of fungal infection and peaked at 48 h (Figure 2b). Compared with the expression levels of *FoUpe9* in fungal conidia or mycelium, *FoUpe9* was remarkably higher in fungal infection stages. These results indicate that *FoUpe9* can be highly induced after induction or in the early stage of fungal infection, suggesting that *FoUpe9* may play an important role in Foc TR4–banana interaction.

### 3.4. FoUpe9 Could Inhibit Plant Immune Responses in Nicotiana benthamiana

**Figure 3:**
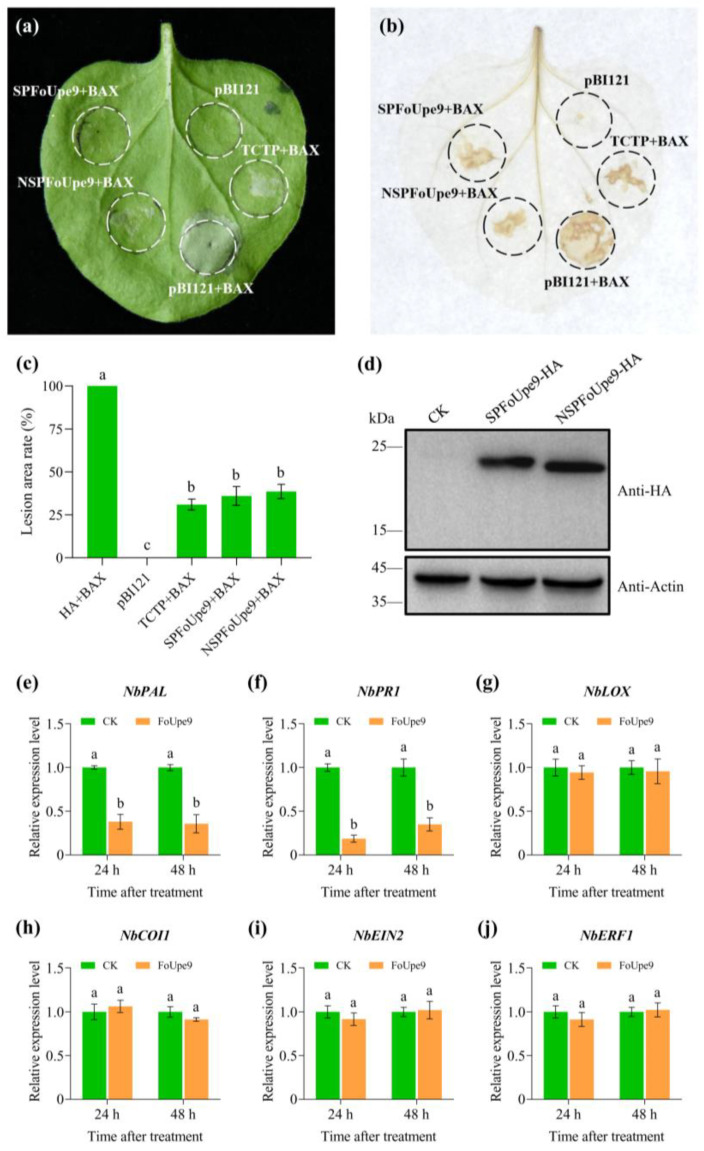
FoUpe9 suppressed plant immune responses in *N. benthamiana*. (**a**) FoUpe9 inhibited the cell death triggered by BAX in *N. benthamiana*. The *Agrobacterium* GV3101 strain carrying *SPFoUpe9*, *NSPFoUpe9*, *TCTP*, or pBI121 empty vector was infiltrated into *N. benthamiana* leaves, followed 24 h later with GV3101 strain harboring *BAX*. The images were photographed 4 days after infiltration. (**b**) ROS accumulation in (**a**) was detected by DAB staining. (**c**) The relative lesion areas were measured by ImageJ software. (**d**) Western blot analysis confirming protein expression with an anti-HA tag antibody using protein from *N. benthamiana* leaves. Actin was used as an internal reference. (**e**–**j**), Relative expression levels of four defense-related genes, *NbPAL* (**e**), *NbPR1* (**f**), *NbLOX* (**g**), *NbCOI1* (**h**), *NbEIN2* (**i**), and *NbERF1* (**j**) in *N. benthamiana* leaves were measured by RT-qPCR. *NbEF1α* was used as an internal reference. Values are the means based on three independent experiments, and bars indicate standard deviations. Different letters indicate statistical significance (*p* < 0.05) using Duncan’s new multiple-range method.

The full length of the FoUpe9 sequence with the signal peptide (SPFoUpe9) or without the signal peptide (NSPFoUpe9) was separately cloned into the pBI121-HA vector, and the recombinant vectors were transformed into *Agrobacterium tumefaciens*. SPFoUpe9 and NSPFoUpe9 were transiently expressed in *N. benthamiana* leaves through *Agrobacterium*-mediated transient expression method. The results showed that both SPFoUpe9 and NSPFoUpe9 could suppress BAX-induced cell death (Figure 3a), but not induce cell death in *N. benthamiana* leaves (Figure S2a). DAB staining also showed that both SPFoUpe9 and NSPFoUpe9 could inhibit BAX-induced ROS accumulation (Figure 3b), but neither of them could induce ROS accumulation in *N. benthamiana* (Figure S2b). In addition, ROS levels calculated by ImageJ 1.54f were also similar to the results of DAB staining (Figure 3c). Western blot assay indicated that the SPFoUpe9-HA and NSPFoUpe9-HA fusion proteins were successfully expressed in *N. benthamiana* leaves (Figure 3d). RT-qPCR analysis further confirmed that FoUpe9 plays a crucial role in regulating the expression of defense-related genes in *N. benthamiana* (Figure 3e–j). The expression of SA signaling-related genes, *NbPAL* and *NbPR1*, decreased significantly in *N. benthamiana* leaves (Figure 3e,f); however, the expression of JA signaling-related genes (*NbLOX* and *NbCOI1*) and ET signaling-related genes (*NbEIN2* and *NbERF1*) showed no significant changes (Figure 3g–j). Collectively, these results indicate that FoUpe9 may suppress plant immunity by regulating ROS production and the SA signaling pathways in *N. benthamiana*.

### 3.5. FoUpe9 Protein Is Localized in the Nucleus and Cytoplasm

**Figure 4:**
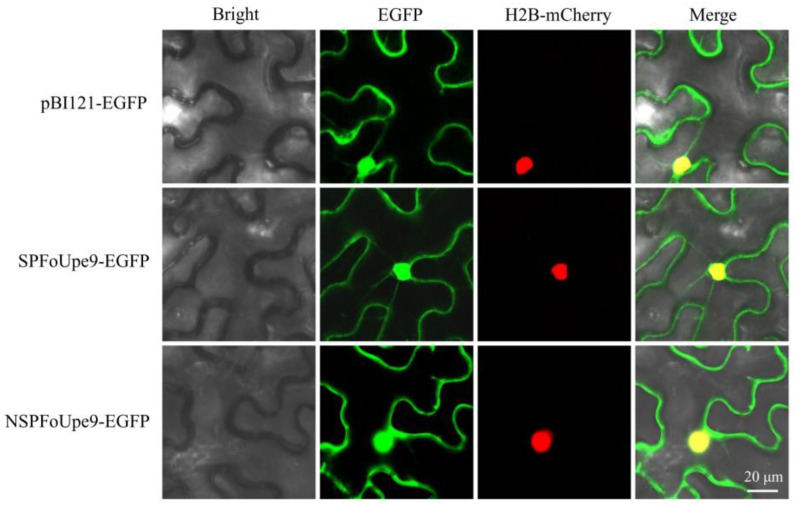
Subcellular localization of FoUpe9 in *N. benthamiana*. Leaves were collected 2 days after agroinfiltration. A laser scanning confocal microscope exhibits the subcellular distribution of SPFoUpe9-EGFP fusion protein and NSPFoUpe9-EGFP in H2B-mCherry transgenic *N. benthamiana*. H2B fusion with the mCherry protein was used as a nuclear location marker. Scale bars represent 20 μm.

To determine the subcellular localization of the FoUpe9 protein, FoUpe9 with or without the signal peptide (SPFoUpe9 and NSPFoUpe9) was separately fused with an enhanced green fluorescent protein (EGFP) in pBI121, then transiently expressed in H2B-mCherry transgenic *N. benthamiana* leaves by agroinfiltration. Confocal fluorescence microscopy showed that fluorescent signals for cells expressing EGFP alone were found in the nucleus and cytoplasm, whereas the signals for cells expressing mCherry existed in the nucleus (Figure 4). The signals for SPFoUpe9-EGFP and NSPFoUpe9-EGFP were detected in both the cytoplasm and the nucleus of *N. benthamiana* cells (Figure 4), indicating FoUpe9 targeted the cytoplasm and nucleus in plant cells.

### 3.6. FoUpe9 Is Dispensable in Mycelial Growth and Conidiation

**Figure 5:**
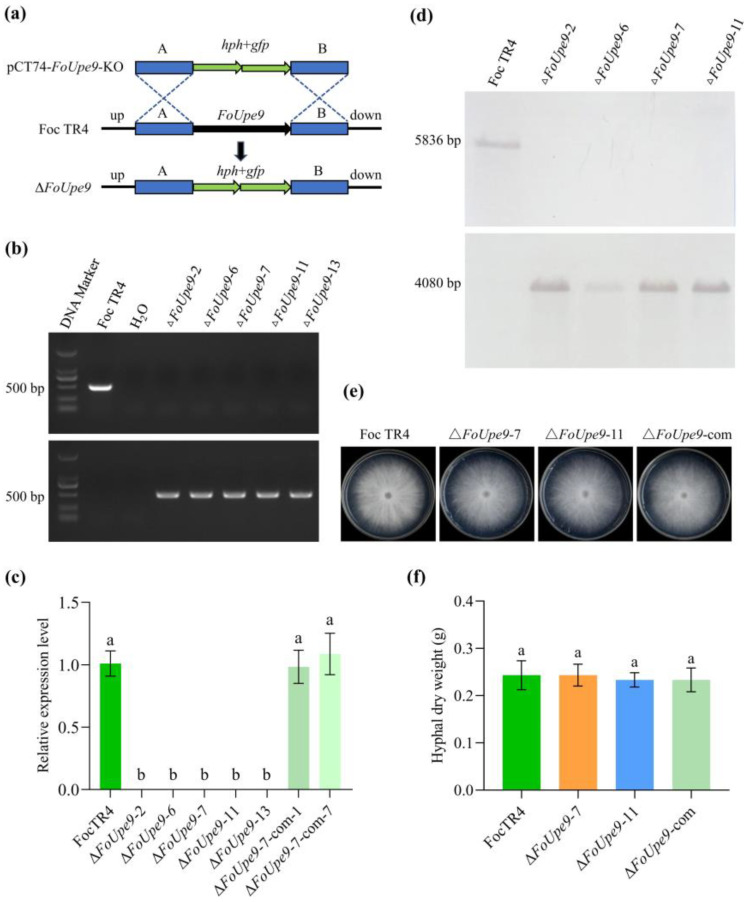
Generation of the *FoUpe9* mutants. (**a**) Approach for *FoUpe9* gene deletion by homologous recombination. Up, upstream homologous arms; Down, downstream homologous arms. (**b**) PCR analysis using *FoUpe9* (upper) and *hph* (lower) as probes. (**c**) RT-qPCR analysis of *FoUpe9* expression in the deletion mutants and complementation strains. (**d**) Southern blot confirmation using *FoUpe9* (upper) and *hph* gene (lower) as probes. (**e**) Colony morphology on PDA media. Photographs were taken after inoculation for 6 days. (**f**) Hyphal dry weight. Hyphae were collected after 2 days cultured in CM media. Values are the means based on three independent experiments, and bars indicate standard deviations. Different letters indicate statistical significance (*p* < 0.05) using Duncan’s new multiple-range method.

To examine the biological function of *FoUpe9* in Foc TR4, we used the homologous recombination method to knock out the *FoUpe9* gene in the wild-type strain (Figure 5a). Through hygromycin screening, we obtained 27 transformants preliminarily. Five mutants were successfully verified to contain the *hph* gene and lack the *FoUpe9* gene by PCR and RT-qPCR analysis (Figure 5b,c). Four deletion mutants (Δ*FoUpe9*-2, Δ*FoUpe9*-6, Δ*FoUpe9*-7, and Δ*FoUpe9*-11) were further confirmed by southern blot assay using both *FoUpe9*-specific and *hph*-specific probes (Figure 5d). Then, we reintroduced the *FoUpe9* gene into the Δ*FoUpe9*-7 deletion mutant and obtained 18 transformants through zeocin resistance selection. After PCR and RT-qPCR verification, four complementation strains (Δ*FoUpe9*-7-com-1, Δ*FoUpe9*-7-com-7, Δ*FoUpe9*-7-com-12, and Δ*FoUpe9*-7-com-15) were confirmed to be correct (Figure S3a,b). There were no differences in the colony morphology and mycelial growth rate among the WT strain, deletion mutants, and complementation strains on PDA (Figure 5e), CM (Figure S4a), and MM plates (Figure S4b). Therefore, Δ*FoUpe9*-7 and Δ*FoUpe9*-11 were selected as the representatives of the deletion mutants, and Δ*FoUpe9*-7-com-1 was selected as the representative of the complementation strains (named Δ*FoUpe9*-com). Additionally, no obvious differences were observed in conidial morphology (Figure S5a), mycelial morphology (Figure S5b), mycelial dry weight (Figure 5f), conidiation (Figure S6a), and conidial germination rate (Figure S6b). These results suggest that *FoUpe9* has no significant effect on mycelial growth and conidiation in Foc TR4.

### 3.7. FoUpe9 Has No Effect on Sensitivity to Various Stresses

To test whether *FoUpe9* is involved in stress tolerance, NaCl and sorbitol were used to mimic osmotic stress, H_2_O_2_ was used to simulate oxidative stress, while SDS, CFW, and CR were used to induce cell-wall integrity stress. There were no differences in the colony morphology or mycelial growth rate among the WT strain, Δ*FoUpe9* strains, and Δ*FoUpe9*-com strains under the aforementioned stress conditions (Figure S7a,b). These findings suggest that *FoUpe9* has no effect on sensitivity to different stresses in Foc TR4.

### 3.8. FoUpe9 Is Essential for the Full Virulence of Foc TR4

**Figure 6:**
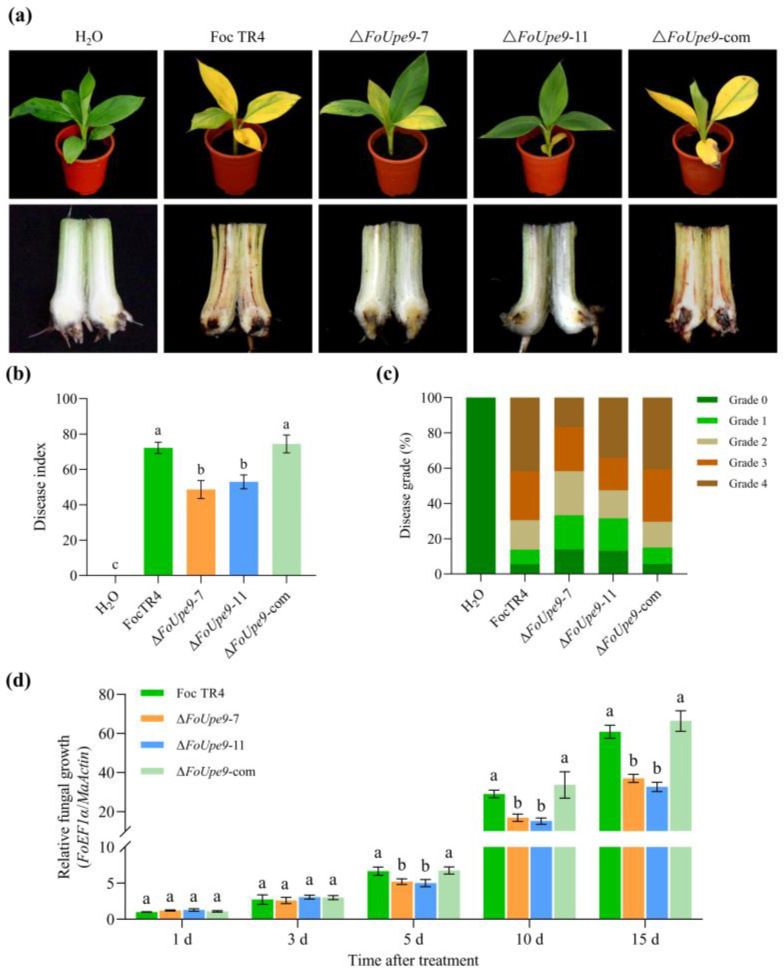
*FoUpe9* deletion attenuated Foc TR4 virulence. (**a**) Pathogenicity assays on banana seedlings inoculated with the conidial suspension of WT, *FoUpe9* deletion mutants, and complemented strains and imaged 21 days after inoculation. (**b**) Disease index. (**c**) Disease grade. (**d**) Relative fungal biomass in the inoculated roots. Values are the means based on three independent experiments, and bars indicate standard deviations. Different letters indicate statistical significance (*p* < 0.05) using Duncan’s new multiple-range method.

To determine the possible function of *FoUpe9* in Foc TR4 virulence, the Brazilian seedlings were inoculated with conidia of the WT, Δ*FoUpe9*, and Δ*FoUpe9*-com strains. Obvious disease symptoms such as leaf yellowing, pseudostem browning, and wilting were observed in the banana seedlings inoculated with the WT and ∆*FoUpe9*-com strains, whereas significantly reduced disease symptoms were detected in the banana seedlings inoculated with the two ∆*FoUpe9* strains (Figure 6a). Consistent with the symptom observation, the disease index of ∆*FoUpe9*-inoculated banana plants was significantly lower than that of the WT- and Δ*FoUpe9*-com-inoculated plants (Figure 6b). The disease symptom severity of the banana seedlings inoculated with the ∆*FoUpe9* strains was also significantly delayed compared with that of the seedlings inoculated with the WT and ∆*FoUpe9*-com strains (Figure 6c). To further determine the function of *FoUpe9* in virulence, we quantified the relative fungal biomass of Foc TR4 in banana roots inoculated by all the tested strains. Compared with the plants inoculated with the WT and ∆*FoUpe9*-com strains, the plants inoculated with ∆*FoUpe9* strains led to a significant decrease in fungal biomass in planta (Figure 6d). These results show that *FoUpe9* deletion results in attenuated virulence of Foc TR4 to banana, confirming the critical role of *FoUpe9* in Foc TR4 virulence.

### 3.9. FoUpe9 Suppressed ROS Accumulation and Immune Response in Banana Plants

**Figure 7:**
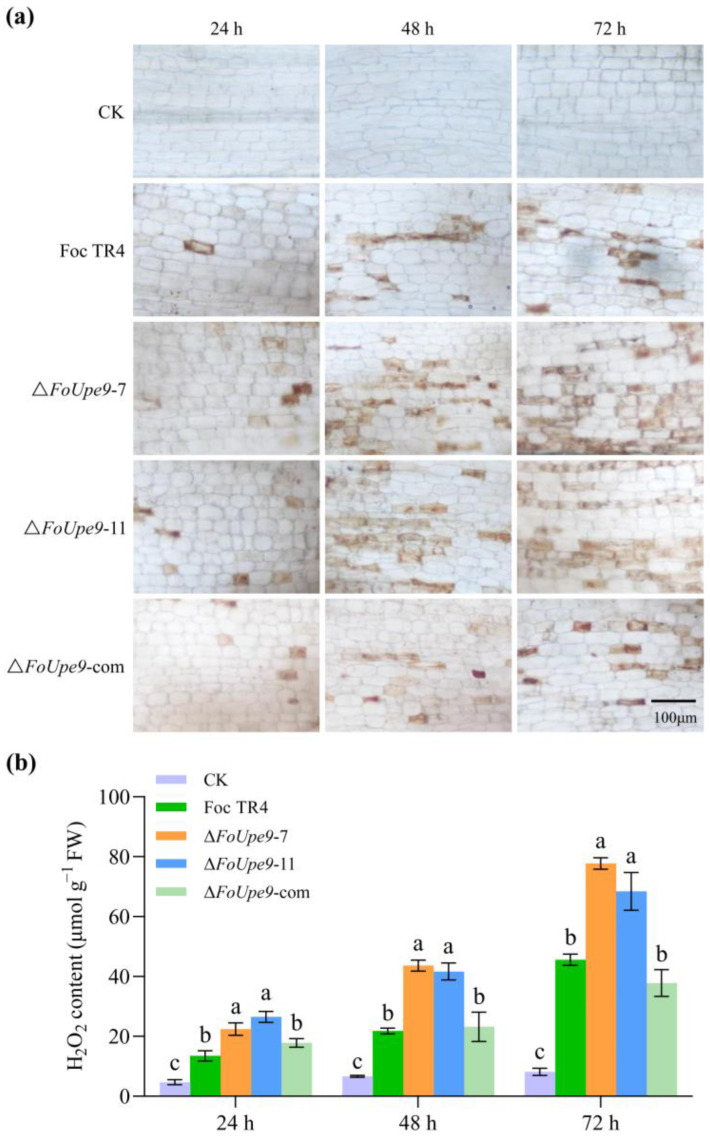
FoUpe9 could inhibit ROS accumulation in banana roots. (**a**) ROS production was determined by DAB staining. (**b**) Quantitative analysis of H_2_O_2_ content. Experiments were repeated three times. Values are the means based on three independent experiments, and bars indicate standard deviations. Different letters indicate statistical significance (*p* < 0.05) using Duncan’s new multiple-range method.

To evaluate whether the reduced virulence of *FoUpe9* deletion mutants is related to the plant immune responses, we first conducted qualitative and quantitative analysis to test the ROS production. DAB staining showed a higher increase in H_2_O_2_ accumulations in Δ*FoUpe9*-inoculated banana plants compared with the WT- and ∆ *FoUpe9*-com-inoculated plants (Figure 7a). Consistent with the histochemical staining results, quantitative detection of H_2_O_2_ contents also showed that ∆*FoUpe9* inoculation led to higher ROS contents than WT- and ∆*FoUpe9*-com-inoculation (Figure 7b).

**Figure 8:**
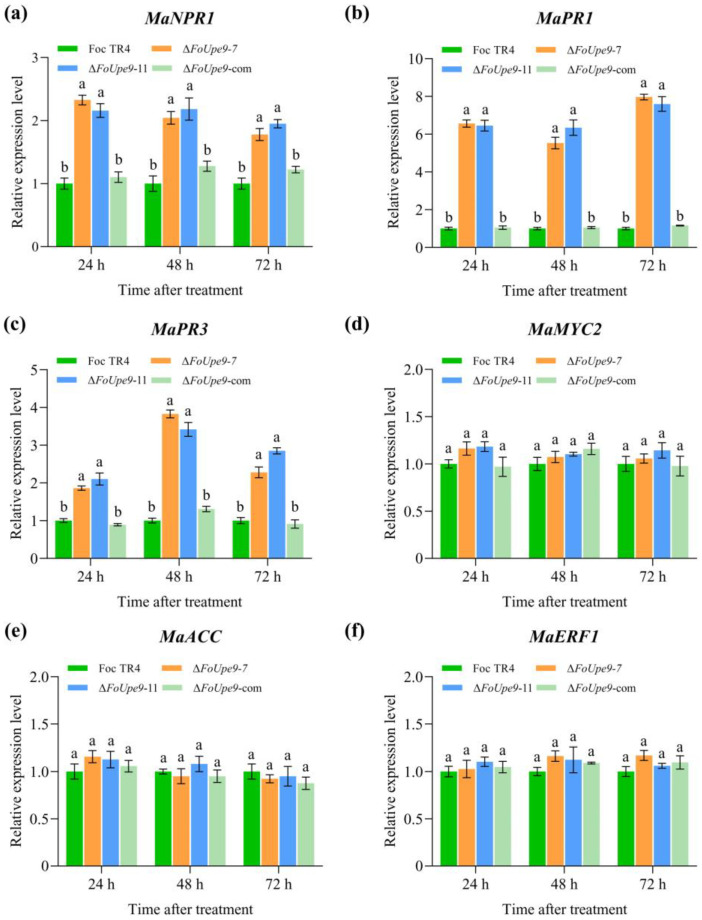
Expression patterns of six defense-related genes in banana seedlings after inoculation determined by RT-qPCR. (**a**) *MaNPR1*; (**b**) *MaPR1*; (**c**) *MaPR3*; (**d**) *MaMYC2*; (**e**) *MaACC*; (**f**) *MaERF1*. The *MaActin* gene was used as the internal reference. Values are the means based on three independent experiments, and bars indicate standard deviations. Different letters indicate statistical significance (*p* < 0.05) using Duncan’s new multiple-range method.

Furthermore, we further measured the expression levels of the defense marker genes for salicylic acid (SA), jasmonic acid (JA), and ethylene (ET) signaling pathways in banana plants through RT-qPCR. The results showed that the expression of the SA signaling marker genes (*MaNPR1*, *MaPR1*, and *MaPR3*) were remarkably increased in Δ*FoUpe9*-inoculated banana plants at 24 h, 48 h, and 72 h post-inoculation, compared to that of WT- and ∆*FoUpe9*-com-inoculated plants (Figure 8a–c). However, the expression of the JA signaling marker genes (*MaMYC2* and *MaACC*) and the ET signaling marker gene (*MaERF1*) showed no significant changes in the WT-, Δ*FoUpe9-*, and Δ*FoUpe9*-com-inoculated plants (Figure 8d–f).

## 4. Discussion

Fungal pathogens secrete various effector proteins to suppress plant immunity and support their infection [31]. A lot of secreted proteins from different phytopathogenic fungi have been characterized as effectors, and participate in manipulating plant immunity [32,33,34,35]. Effectors facilitate the virulence of the phytopathogens by modulating host functions including cell-wall composition and intracellular signaling [36,37,38,39]. However, the substantial diversity and quantity of effectors lead to various ways of function; most of their mechanisms remain unknown, and need to be identified urgently [40,41,42]. Banana Fusarium wilt caused by Foc TR4 is a highly damaging disease worldwide [43]. Recently, evidence emerged that secreted proteins can act as pathogenicity factors and play important roles in the Foc TR4–banana interactions [17,44]. However, most effectors do not contain any recognizable domains or functional annotations, which may represent novel effectors [45]. Therefore, further investigations of these new candidate effectors are needed, which will unveil the new molecular mechanisms of Foc TR4 pathogenesis.

In our previous study, we performed a shotgun-based secretome analysis of Foc TR4 and predicted 70 candidate effectors using bioinformatic approaches [17]. Among these, only a few effectors had been well characterized, including SIX8, FSE1, FocRnt2, and FoCupin1 [19,20,21,22]. Many of these candidate effectors are small, secreted proteins without any known domains, and the molecular mechanisms underlying interaction between Foc TR4 and bananas are still unclear [46]. In this study, we characterized an uncharacterized protein (named FoUpe9) without any known domain or functional annotation from the Foc TR4 secretome. Phylogenetic tree analysis and amino acid sequence alignments showed that FoUpe9 is widely present in different plant pathogenic fungi and highly conserved among *Fusarium* species (Figure 1). However, the sequence identity between FoUpe9 and the orthologs from other photopathogenic fungi varies greatly, such as sharing only 43.13% with uncharacterized protein (KAI9904398.1) from *Trichothecium roseum* and 41.55% with uncharacterized protein (XP_009649295.1) from *Verticillium dahliae*, probably reflecting a result of rapid evolution. FoUpe9 contains a functional secretory which was confirmed by a yeast signal sequence trap system (Figure 1). Subcellular localization showed that FoUpe9 targeted the cytoplasm and nucleus in *N. benthamiana* (Figure 4). A similar phenomenon was also observed in several effectors of phytopathogenic fungi, such as FSE1, FoSSP17, and FocRnt2 from Foc4 [19,22,47], PpE4 from *Phytophthora parasitica*, and PlAvh142 from *Peronophythora litchii* [48,49]. RT-qPCR analysis showed that *FoUpe9* is highly expressed after induction by banana extracts in vitro or at the early infection stages of Foc TR4 (Figure 2), suggesting that *FoUpe9* may play a vital role in Foc TR4–banana interaction. Meanwhile, transient expression of *FoUpe9* could suppress BAX-induced cell death and ROS production in *N. benthamiana* leaves (Figure 3). Similar with our results, several effectors of Foc TR4, such as FoCupin1, FocM35_1, FoSSP17, and FocRnt2, suppress BAX-triggered cell death in leaves of *N. benthamiana* [21,22,44,47]. However, neither SPFoUpe9 nor NSPFoUpe9 could induce cell death or ROS production (Figure S2), suggesting that the signal peptide of FoUpe9 may be unnecessary for its host immunity-inducing ability in *N. benthamiana*. Therefore, our study demonstrated that FoUpe9 may inhibit plant immune responses, thus enhancing Foc TR4 virulence.

To further characterize the function of *FoUpe9* in Foc TR4, we generated *FoUpe9* deletion mutants and the complementation mutants. The deletion of *FoUpe9* did not affect mycelial growth, conidiation, spore germination, or the sensitivity to various stresses (Figure 5), suggesting that *FoUpe9* does not affect the maturation and development of Foc TR4. However, deletion of the *FoUpe9* remarkably attenuated the virulence of Foc TR4 and decreased the fungal colonization in planta (Figure 6), indicating that *FoUpe9* may play an important role in Foc TR4 pathogenicity. Additionally, we found that FoUpe9 can inhibit ROS accumulation (Figure 7) and the expression of SA-related defense genes (Figure 8), further revealing a negative impact on plant immunity. Consistent with these results, FoUpe9 also remarkably down-regulated the expression of SA signaling-related genes (*NbPAL* and *NbPR1*) in *N. benthamiana* (Figure 3). Similar results have been reported that an effector FoCupin1 in Foc TR4 is able to inhibit ROS accumulation, decrease the expression of defense marker genes, and suppress plant defense responses in the non-host *N. benthamiana* and the host banana cultivar Brazilian [21]. FoSSP71, a novel effector protein in Foc4, is an essential virulence factor, which enhances pathogenicity by suppressing host immune responses, including ROS bursts, callose deposition, and defense-related gene expression [50]. The effector FocRnt2 also suppressed ROS accumulation, compromised fungal infection, up-regulated the expression of defense-related genes in banana plants, and played key roles in fungal virulence [22]. The effector FocM35_1 was found to suppress plant immunity and play an important role in Foc TR4 virulence [44]. Furthermore, the precise molecular mechanism of how FoUpe9 functions in Foc TR4–banana interaction needs to be further elucidated.

Phytopathogenic fungi deliver effector proteins directly into living plant cells to suppress defenses and enable pathogens to rapidly invade and proliferate within plant tissue. However, little is known about the mechanisms by which these pathogens translocate effector proteins across the plasma membrane into the plant cytoplasm [51]. Recent studies showed that effectors may be evolutionarily conserved or unique for different phytopathogenic fungi [52,53]. For example, the rice blast fungus *Magnaporthe oryzae* forms a specialized interfacial region known as the biotrophic interfacial complex (BIC), which is necessary for effector delivery into plant cells. Oliveira-Garcia et al. [52] further provides strong evidence that clathrin-mediated endocytosis is necessary for effector translocation into plant cells at the BIC. The oomycete pathogen *Phytophthora infestans* has a BFA-insensitive, Golgi-independent secretion system for cytoplasmic effectors, which usually contains amino acid translocation motifs [54]. In this study, our results showed that transient expression of *FoUpe9* inhibited BAX-induced cell death in *N. benthamiana*, indicating that FoUpe9 performed cell-death-suppressive functions inside plant cells. Subcellular localization showed that FoUpe9 targeted the nucleus and cytoplasm in plant cells. How FoUpe9 enters plant cells remains a topic for further study.

In summary, we identified and characterized a novel effector FoUpe9 in Foc TR4 that is highly conserved in *Fusarium* species and targets the nucleus and cytoplasm in plant cells. FoUpe9 suppresses plant immunity in the host and non-host plants by weakening ROS accumulation and the SA-mediated defense responses, thus facilitating fungal infection and promoting fungal virulence during the early stage of banana–Foc TR4 interaction. However, further studies on the exact mechanisms of plant defense response regulated by FoUpe9 are also needed.

## Data Availability

The original contributions presented in this study are included in the article/Supplementary Materials. Further inquiries can be directed to the corresponding authors.

## Supplementary Materials

The following supporting information can be downloaded at https://www.mdpi.com/article/10.3390/jof11040308/s1, Figure S1: Amino acid sequence alignment of FoUpe9 and its homologs in other species; Figure S2: FoUpe9 could not induce cell death and ROS accumulation in *N. benthamiana*; Figure S3: PCR and RT-qPCR confirmation of four *FoUpe9* complementation strains; Figure S4: FoUpe9 is not essential for the mycelial growth of Foc TR4; Figure S5: FoUpe9 is not essential for the conidiation of Foc TR4; Figure S6: FoUpe9 is not essential for the conidial morphology and mycelial morphology of Foc TR4; Figure S7: FoUpe9 is not sensitive to various stresses; Table S1: Primers used in this study. References [22,55,56,57,58] are cited in the Supplementary Materials.

## Abbreviations

The following abbreviations are used in this manuscript:

BAX	Bcl2-associated X protein
CFW	Calcofluor white
CR	Congo red
EGFP	Enhanced green fluorescent protein
*hph*	*Hygromycin B phosphotransferase*
PEG	Polyethylene glycol
ROS	Reactive oxygen species
SA	Salicylic acid
TCTP	Translationally controlled tumor protein
TTC	2,3,5-triphenyl tetrazolium chloride
YPDA	Yeast peptone dextrose adenine

## References

[1] Ploetz R.C. (2015). Fusarium Wilt of Banana. Phytopathology.

[2] Pegg K.G., Coates L.M., O’Neill W.T., Turner D.W. (2019). The Epidemiology of Fusarium Wilt of Banana. Front. Plant Sci..

[3] Bragard C., Baptista P., Chatzivassiliou E., Di Serio F., Gonthier P., Jaques Miret J.A., Justesen A.F., Macleod A., Magnusson C.S., Milonas P. (2022). Pest categorisation of *Fusarium oxysporum* f. sp. *cubense* tropical race 4. EFSA J..

[4] Li W., Wang X., Li C., Sun J., Li S., Peng M. (2019). Dual species transcript profiling during the interaction between banana (*Musa acuminata*) and the fungal pathogen *Fusarium oxysporum* f. sp. *cubense*. BMC Genom..

[5] Dong H., Fan H., Lei Z., Wu C., Zhou D., Li H. (2019). Histological and gene expression analyses in banana reveals the pathogenic differences between races 1 and 4 of banana Fusarium wilt pathogen. Phytopathology.

[6] Zuriegat Q., Zheng Y., Liu H., Wang Z., Yun Y. (2021). Current progress on pathogenicity-related transcription factors in *Fusarium oxysporum*. Mol. Plant Pathol..

[7] Ordonez N., Seidl M.F., Waalwijk C., Drenth A., Kilian A., Thomma B.P.H.J., Ploetz R.C., Kema G.H.J. (2015). Worse comes to worst: Bananas and Panama disease—When plant and pathogen clones meet. PLoS Pathog..

[8] Thangavelu R., Amaresh H., Gopi M., Loganathan M., Nithya B., Ganga Devi P., Anuradha C., Thirugnanavel A., Patil K.B., Blomme G. (2024). Geographical distribution, host range and genetic diversity of *Fusarium oxysporum* f. sp. *cubense* causing Fusarium wilt of banana in India. J. Fungi.

[9] Yang D., Du C., Zhang J., Pan L., Wei S., Jiang S., Li C., San C.C., Huy N.D., Ye Y. (2023). Validation and application of a molecular detection system for Fusarium wilt of banana in China. Plant Dis..

[10] Wang C., Zheng Y., Liu Z., Qian Y., Li Y., Yang L., Liu S., Liang W., Li J. (2023). The secreted FolAsp aspartic protease facilitates the virulence of *Fusarium oxysporum* f. sp. *lycopersici*. Front. Microbiol..

[11] Kim C., Song H., Lee Y. (2022). Ambivalent response in pathogen defense: A double-edged sword?. Plant Commun..

[12] Wang Y., Pruitt R.N., Nurnberger T., Wang Y. (2022). Evasion of plant immunity by microbial pathogens. Nat. Rev. Microbiol..

[13] Qiu X., Kong L., Chen H., Lin Y., Tu S., Wang L., Chen Z., Zeng M., Xiao J., Yuan P. (2023). The *Phytophthora sojae* nuclear effector PsAvh110 targets a host transcriptional complex to modulate plant immunity. Plant Cell.

[14] Jones J.D.G., Staskawicz B.J., Dangl J.L. (2024). The plant immune system: From discovery to deployment. Cell.

[15] Jones J.D.G., Dangl J.L. (2006). The plant immune system. Nature.

[16] Ngou B.P.M., Ding P., Jones J.D.G. (2022). Thirty years of resistance: Zig-zag through the plant immune system. Plant Cell.

[17] He Y., Zhou X., Li J., Li H., Li Y., Nie Y. (2021). In Vitro secretome analysis suggests differential pathogenic mechanisms between *Fusarium oxysporum* f. sp. *cubense* race 1 and race 4. Biomolecules.

[18] Guo L., Wang J., Liang C., Yang L., Zhou Y., Liu L., Huang J. (2022). Fosp9, a novel secreted protein, is essential for the full virulence of *Fusarium oxysporum* f. sp. *cubense* on banana (*Musa* spp.). Appl. Environ. Microbiol..

[19] Yang Y., An B., Guo Y., Luo H., He C., Wang Q. (2023). A novel effector, FSE1, regulates the pathogenicity of *Fusarium oxysporum* f. sp. *cubense* tropical race 4 to banana by targeting the MYB transcription factor MaEFM-like. J. Fungi.

[20] An B., Hou X., Guo Y., Zhao S., Luo H., He C., Wang Q. (2019). The effector SIX8 is required for virulence of *Fusarium oxysporum* f.sp. *cubense* tropical race 4 to *Cavendish banana*. Fungal Biol..

[21] Yan T., Zhou X., Li J., Li G., Zhao Y., Wang H., Li H., Nie Y., Li Y. (2022). FoCupin1, a Cupin_1 domain-containing protein, is necessary for the virulence of *Fusarium oxysporum* f. sp. *cubense* tropical race 4. Front. Microbiol..

[22] He Y., Li P., Zhou X., Ali S., Zhu J., Ma Y., Li J., Zhang N., Li H., Li Y. (2024). A ribonucleaseT2 protein FocRnt2 contributes to the virulence of *Fusarium oxysporum* f. sp. *cubense* tropical race 4. Mol. Plant Pathol..

[23] Lyu X., Shen C., Fu Y., Xie J., Jiang D., Li G., Cheng J. (2016). A small secreted virulence-related protein is essential for the necrotrophic interactions of *Sclerotinia sclerotiorum* with its host plants. PLoS Pathog..

[24] Liu X., Gao Y., Guo Z., Wang N., Wegner A., Wang J., Zou X., Hu J., Liu M., Zhang H. (2022). MoIug4 is a novel secreted effector promoting rice blast by counteracting host OsAHL1-regulated ethylene gene transcription. New Phytol..

[25] Li G., Shi Q., He Y., Zhu J., Zhong M., Tong L., Li H., Nie Y., Li Y. (2023). Screening of candidate effectors from *Magnaporthe oryzae* by in vitro secretomic analysis. Int. J. Mol. Sci..

[26] Yin W., Wang Y., Chen T., Lin Y., Luo C. (2018). Functional evaluation of the signal peptides of secreted proteins. Bio-Protocol.

[27] Livak K.J., Schmittgen T.D. (2001). Analysis of relative gene expression data using real-time quantitative PCR and the 2(-Delta Delta C(T)) Method. Methods.

[28] Ma L., Lukasik E., Gawehns F., Takken F.L.W. (2012). The use of agroinfiltration for transient expression of plant resistance and fungal effector proteins in *Nicotiana benthamiana* leaves. Methods Mol. Biol..

[29] Dai Y., Cao Z., Huang L., Liu S., Shen Z., Wang Y., Wang H., Zhang H., Li D., Song F. (2016). CCR4-not complex subunit Not2 plays critical roles in vegetative growth, conidiation and virulence in watermelon Fusarium wilt pathogen *Fusarium oxysporum* f. sp. *niveum*. Front. Microbiol..

[30] Brennan T., Frenkel C. (1977). Involvement of hydrogen peroxide in the regulation of senescence in pear. Plant Physiol..

[31] Chen X., Duan Y., Qiao F., Liu H., Huang J., Luo C., Chen X., Li G., Xie K., Hsiang T. (2022). A secreted fungal effector suppresses rice immunity through host histone hypoacetylation. New Phytol..

[32] Kim K., Jeon J., Choi J., Cheong K., Song H., Choi G., Kang S., Lee Y. (2016). Kingdom-wide analysis of fungal small secreted proteins (SSPs) reveals their potential role in host association. Front. Plant Sci..

[33] Ma M., Tang L., Sun R., Lyu X., Xie J., Fu Y., Li B., Chen T., Lin Y., Yu X. (2024). An effector SsCVNH promotes the virulence of *Sclerotinia sclerotiorum* through targeting classIII peroxidase AtPRX71. Mol. Plant Pathol..

[34] Hoang C.V., Bhaskar C.K., Ma L. (2021). A Novel Core Effector Vp1 Promotes Fungal colonization and virulence of *Ustilago maydis*. J. Fungi.

[35] Yang B., Wang Y., Tian M., Dai K., Zheng W., Liu Z., Yang S., Liu X., Shi D., Zhang H. (2021). Fg12 ribonuclease secretion contributes to *Fusarium graminearum* virulence and induces plant cell death. J. Integr. Plant Biol..

[36] Redkar A., Sabale M., Schudoma C., Zechmann B., Gupta Y.K., López-Berges M.S., Venturini G., Gimenez-Ibanez S., Turrà D., Solano R. (2022). Conserved secreted effectors contribute to endophytic growth and multihost plant compatibility in a vascular wilt fungus. Plant Cell.

[37] Bindics J., Khan M., Uhse S., Kogelmann B., Baggely L., Reumann D., Ingole K.D., Stirnberg A., Rybecky A., Darino M. (2022). Many ways to TOPLESS—manipulation of plant auxin signalling by a cluster of fungal effectors. New Phytol..

[38] Shang S., Liang X., Liu G., Du Y., Zhang S., Meng Y., Zhu J., Rollins J.A., Zhang R., Sun G. (2024). A fungal effector suppresses plant immunity by manipulating DAHPS-mediated metabolic flux in chloroplasts. New Phytol..

[39] Shang S., Liu G., Zhang S., Liang X., Zhang R., Sun G. (2024). A fungal CFEM-containing effector targets NPR1 regulator NIMIN2 to suppress plant immunity. Plant Biotechnol. J..

[40] Zhang M., Xie S., Zhao Y., Meng X., Song L., Feng H., Huang L. (2019). Hce2 domain-containing effectors contribute to the full virulence of *Valsa mali* in a redundant manner. Mol. Plant Pathol..

[41] Yang B., Yang S., Guo B., Wang Y., Zheng W., Tian M., Dai K., Liu Z., Wang H., Ma Z. (2021). The *Phytophthora* effector Avh241 interacts with host NDR1-like proteins to manipulate plant immunity. J. Integr. Plant Biol..

[42] Chen X., Pan S., Bai H., Fan J., Batool W., Shabbir A., Han Y., Zheng H., Lu G., Lin L. (2023). A nonclassically secreted effector of *Magnaporthe oryzae* targets host nuclei and plays important roles in fungal growth and plant infection. Mol. Plant Pathol..

[43] Dita M., Barquero M., Heck D., Mizubuti E.S.G., Staver C.P. (2018). Fusarium wilt of banana: Current knowledge on epidemiology and research needs toward sustainable disease management. Front. Plant Sci..

[44] Zhang X., Huang H., Wu B., Xie J., Viljoen A., Wang W., Mostert D., Xie Y., Fu G., Xiang D. (2021). The M35 metalloprotease effector FocM35_1 is required for full virulence of *Fusarium oxysporum* f. sp. *cubense* tropical race 4. Pathogens.

[45] Wang Y., Zhang X., Wang T., Zhou S., Liang X., Xie C., Kang Z., Chen D., Zheng L. (2022). The small secreted protein FoSsp1 elicits plant defenses and negatively regulates pathogenesis in *Fusarium oxysporum* f. sp. *cubense* (Foc4). Front. Plant Sci..

[46] Zhao S., An B., Guo Y., Hou X., Luo H., He C., Wang Q. (2020). Label free proteomics and systematic analysis of secretome reveals effector candidates regulated by SGE1 and FTF1 in the plant pathogen *Fusarium oxysporum* f. sp. *cubense* tropical race 4. BMC Genom..

[47] Wang T., Xu Y., Zhao Y., Liang X., Liu S., Zhang Y., Kang Z., Chen D., Zheng L. (2023). Systemic screening of *Fusarium oxysporum* candidate effectors reveals FoSSP17 that suppresses plant immunity and contributes to virulence. Phytopathol. Res..

[48] Situ J., Jiang L., Fan X., Yang W., Li W., Xi P., Deng Y., Kong G., Jiang Z. (2020). An RXLR effector PlAvh142 from *Peronophythora litchii* triggers plant cell death and contributes to virulence. Mol. Plant Pathol..

[49] Huang G., Liu Z., Gu B., Zhao H., Jia J., Fan G., Meng Y., Du Y., Shan W. (2019). An RXLR effector secreted by *Phytophthora parasitica* is a virulence factor and triggers cell death in various plants. Mol. Plant Pathol..

[50] Liu S., Wu J., Sun Y., Xu Y., Zhou S., Luo P., Wang Z., Chen D., Liang X., Kang Z. (2025). A novel key virulence factor, FoSSP71, inhibits plant immunity and promotes pathogenesis in *Fusarium oxysporum* f. sp. *cubense*. Microbiol. Spectr..

[51] Lo Presti L., Lanver D., Schweizer G., Tanaka S., Liang L., Tollot M., Zuccaro A., Reissmann S., Kahmann R. (2015). Fungal effectors and plant susceptibility. Annu. Rev. Plant Biol..

[52] Oliveira-Garcia E., Tamang T.M., Park J., Dalby M., Martin-Urdiroz M., Rodriguez Herrero C., Vu A.H., Park S., Talbot N.J., Valent B. (2023). Clathrin-mediated endocytosis facilitates the internalization of *Magnaporthe oryzae* effectors into rice cells. Plant Cell.

[53] Lo Presti L., Kahmann R. (2017). How filamentous plant pathogen effectors are translocated to host cells. Curr. Opin. Plant Biol..

[54] Wang S., Boevink P.C., Welsh L., Zhang R., Whisson S.C., Birch P.R.J. (2017). Delivery of cytoplasmic and apoplastic effectors from Phytophthora infestans haustoria by distinct secretion pathways. New Phytol..

[55] Dalio R.J., Maximo H.J., Roma-Almeida R., Barretta J.N., José E.M., Vitti A.J., Blachinsky D., Reuveni M., Pascholati S.F. (2020). Tea tree oil induces systemic resistance against Fusarium wilt in banana and xanthomonas infection in tomato plants. Plants.

[56] Liu S., Wu B., Yang J., Bi F., Dong T., Yang Q., Hu C., Xiang D., Chen H., Huang H. (2019). A cerato-platanin family protein FocCP1 is essential for the penetration and virulence of *Fusarium oxysporum* f. sp. *cubense* tropical race 4. Int. J. Mol. Sci..

[57] Niu Y., Hu B., Li X., Chen H., Takáč T., Šamaj J., Xu C. (2018). Comparative digital gene expression analysis of tissue-cultured plantlets of highly resistant and susceptible banana cultivarsin response to *Fusarium oxysporum*. Int. J. Mol. Sci..

[58] Zhang L., Ni H., Du X., Wang S., Ma X.W., Nürnberger T., Guo H.S., Hua C. (2017). The Verticillium-specific protein VdSCP7 localizes to the plant nucleus and modulates immunity to fungal infections. New Phytol..

