# Supplementary material for: A Novel Effector FoUpe9 Enhances the Virulence of Fusarium oxysporum f. sp. cubense Tropical Race 4 by Inhibiting Plant Immunity
Source: J Fungi (Basel). 2025 Apr 13;11(4):308. doi: 10.3390/jof11040308 (PMC12028529; doi:10.3390/jof11040308)
Supplement: Supplementary file 1 [file jof-11-00308-s001.zip › Supplementary Information_Table S1_20250317.pdf]

Supplemental Table S1: Primers used in this study

Primer name	Primer Sequence (5'-3')	Reference
Construction of pSUC2 vector for yeast signal trap assay		
pSUC2-*FoUpe9*-F	CGGAATTCATGAAGTTCTTCAGCCTCCTCAC	Present work
pSUC2-*FoUpe9*-R	CCGCTCGAGTCCTGTGACAGTTTGTTGCCG	Present work
Construction of transient expression vector		
pBI121-HA-SP*FoUpe9*-F	GCGTCGACATGAAGTTCTTCAGCCTCCTCAC	Present work
pBI121-HA-SP*FoUpe9*-R	GCTCTAGAGAGATTAAGGGGAGAGATACCGATG	Present work
pBI121-HA-NSP*FoUpe9*-F	GCGTCGACATGACGGCCTCTCCTTTCCGC	Present work
pBI121-HA-NSP*FoUpe9*-R	GCTCTAGAGAGATTAAGGGGAGAGATACCGATG	Present work
Construction of subcellular localization vector		
pBI121-EGFP-SP*FoUpe9*-F	GCGTCGACATGAAGTTCTTCAGCCTCCTCAC	Present work
pBI121-EGFP-SP*FoUpe9*-R	GCTCTAGAGAGATTAAGGGGAGAGATACCGATG	Present work
pBI121-EGFP-NSP*FoUpe9*-F	GCGTCGACATGACGGCCTCTCCTTTCCGC	Present work
pBI121-EGFP-NSP*FoUpe9*-R	GCTCTAGAGAGATTAAGGGGAGAGATACCGATG	Present work
Construction and confirmation of *FoUpe9* deletion and complementation mutants		
FoUpe9-up-F	GGGGTACCACCTCTACAAGGAGCGAGTTAT	Present work
FoUpe9-up-R	GGGGGCCCCCTTCTTGACCACTGACTTGT	Present work
FoUpe9-down-F	CGGAATTCTCTCGGACCACGCCGTT	Present work
FoUpe9-down-R	GCTCTAGATGGAGTGACCAAGTAGGTATGT	Present work
hph-F	TGCTGCTCCATACAAGCCAA	Present work
hph-R	GACATTGGGGAGTTCAGCGA	Present work
FoUpe9-F	GCTAGCACCATTGCTTTTGTCAT	Present work
FoUpe9-R	AGGCTTGGCCAATTCCACAAC	Present work
FoUpe9-com-F	CGGAATTCCTTGACGCAAAGCAACTACG	Present work
FoUpe9-com-R	ATAAGAATGCGGCCGCCGATTCGGATGAACAGC ACC	Present work
FoUpe9-probe-F	GCTAGCACCATTGCTTTTGTCAT	Present work
FoUpe9-probe-R	AGGCTTGGCCAATTCCACAAC	Present work
hph-porobe-F	TGCTGCTCCATACAAGCCAA	Present work
hph-porobe-R	GACATTGGGGAGTTCAGCGA	Present work
RT-qPCR analysis of *FoUpe9* gene in Foc TR4		
q*FoUpe9*-F	CAGTGATAGTAGCACCGCCG	Present work
q*FoUpe9*-R	CAGCGTCACTACAGGAGCAG	Present work
q*FoEF1α*-F	GCTGGTGACTCCAAGAACGA	Liu et al., 2019
q*FoEF1α*-R	CATCTTGACGATGGCGGAGT	Liu et al., 2019
q*FoTubllin*-F	CCTCGTCGATCTTGAGCCTG	Present work
q*FoTubllin*-R	CTGGAAACCCTGGAGGCAAT	Present work

DNA-based qPCR analysis of fungal biomass		
q*FoEF1α*-F	GCTGGTGACTCCAAGAACGA	Liu et al., 2019
q*FoEF1α*-R	CATCTTGACGATGGCGGAGT	Liu et al., 2019
q*MaActin*-F	TGTTGCATCCTGGTACTGCT	Liu et al., 2019
q*MaActin*-R	GGCTTTCTTGCACTGGTACAC	Liu et al., 2019
RT-qPCR analysis of defense-related genes in tobacco and banana		
q*NbEF1α*-F	GGTTAAGATGATGCCGACCAAG	Zhang et al., 2017
q*NbEF1α*-R	CGCCAGTTGGGTCCTTCTTG	Zhang et al., 2017
q*NbPAL*-F	GCTAGATTAGCCCTTGCTTCA	Present work
q*NbPAL*-R	ATTCCTTCCTGCTGTCAGATTAG	Present work
q*NbPR1*-F	CCGCCTTCCCTCAACTCAAC	Zhang et al., 2017
q*NbPR1*-R	GCACAACCAAGACGTACTGAG	Zhang et al., 2017
q*NbERF1*-F	GCTCTTAACGTCGGATGGTC	Zhang et al., 2017
q*NbERF1*-R	AGCCAAACCCTAGCTCCATT	Zhang et al., 2017
q*NbEIN2*-F	CGTCAACTATGCTGAACCATTTG	He et al., 2023
q*NbEIN2*-R	ACGGGCTGCATGGAATTAT	He et al., 2023
q*NbLOX*-F	AAAACCTATGCCTCAAGAAC	Zhang et al., 2017
q*NbLOX*-R	ACTGCTGCATAGGCTTTGG	Zhang et al., 2017
q*NbCOI1*-R	GGCTTGACGTACTTAGGGAAATA	Present work
q*NbCOI1*-R	GGGACACCTTTGCAGTAAGA	Present work
q*MaActin*-F	TGTTGCATCCTGGTACTGCT	Liu et al., 2019
q*MaActin*-R	GGCTTTCTTGCACTGGTACAC	Liu et al., 2019
q*MaPR1*-F	AGGACAACGAGGGGGAGATA	Niu et al., 2018
q*MaPR1*-R	TACGGGTAGGCTGATGGGTT	Niu et al., 2018
q*MaNPR1*-F	GGAGATCCACAAGTAGGTGAAGC	Dalio et al., 2020
q*MaNPR1*-R	AGTCTTGCCAGAGCAACTCG	Dalio et al., 2020
q*MaPR3*-F	GTCACCACCAACATCATCAA	Dalio et al., 2020
q*MaPR3*-R	CCAGCAAGTCGCAGTACCTC	Dalio et al., 2020
q*MaERF1*-F	CCCAAATGTTGGTCCGTTTC	Dalio et al., 2020
q*MaERF1*-R	TCGCTGTCTTCCACGATTCA	Dalio et al., 2020
q*MaACC*-F	GATGCTGCACATCGGCTAGT	Dalio et al., 2020
q*MaACC*-R	GCCACCTGAATACGGCAGAC	Dalio et al., 2020
q*MaMYC2*-F	CGGATCTACCGACGTGGTCT	Dalio et al., 2020
q*MaMYC2*-R	AGCGTCCGGAGAGCTAAAGT	Dalio et al., 2020

References for Supplemental Materials

Dalio, R. J. D., Maximo, H. J., Roma-Almeida, R., Barretta, J. N., Jose, E. M., Vitti, A. J. et al. (2020) Tea tree oil induces systemic resistance against Fusarium wilt in banana and xanthomonas infection in tomato plants. *Plants*, 9, 1137.

He, Y., Li, P., Zhou, X., Ali, S., Zhu, J., Ma, Y. et al. (2023) A ribonuclease T2 protein FocRnt2 contributes to the virulence of *Fusarium oxysporum* f. sp. *cubense* tropical race 4. *Molecular Plant Pathology*, 25, e13502.

Liu, S., Wu, B., Yang, J., Bi, F., Dong, T., Yang, Q. et al. (2019) A cerato-platanin

family protein FocCP1 is essential for the penetration and virulence of *Fusarium oxysporum* f. sp. *cubense* tropical race 4. *International Journal of Molecular Sciences*, 20, 3785.

Niu, Y., Hu, B., Li, X., Chen, H., Takac, T., Samaj, J. et al. (2018) Comparative digital gene expression analysis of tissue-cultured plantlets of highly resistant and susceptible banana cultivarsin response to *Fusarium oxysporum*. *International Journal of Molecular Sciences*, 19, 350.

Zhang, L., Ni, H., Du, X., Wang, S., Ma, X.-W., Nuernberger, T. et al. (2017) The Verticillium-specific protein VdSCP7 localizes to the plant nucleus and modulates immunity to fungal infections. *New Phytologist*, 215 (1), 368-381.
