# Supplementary material for: A Novel Effector FoUpe9 Enhances the Virulence of Fusarium oxysporum f. sp. cubense Tropical Race 4 by Inhibiting Plant Immunity
Source: J Fungi (Basel). 2025 Apr 13;11(4):308. doi: 10.3390/jof11040308 (PMC12028529; doi:10.3390/jof11040308)
Supplement: Supplementary file 1 [file jof-11-00308-s001.zip › Supplementary Information_Figures_JoF_20250317.pdf]

Supplementary Figures

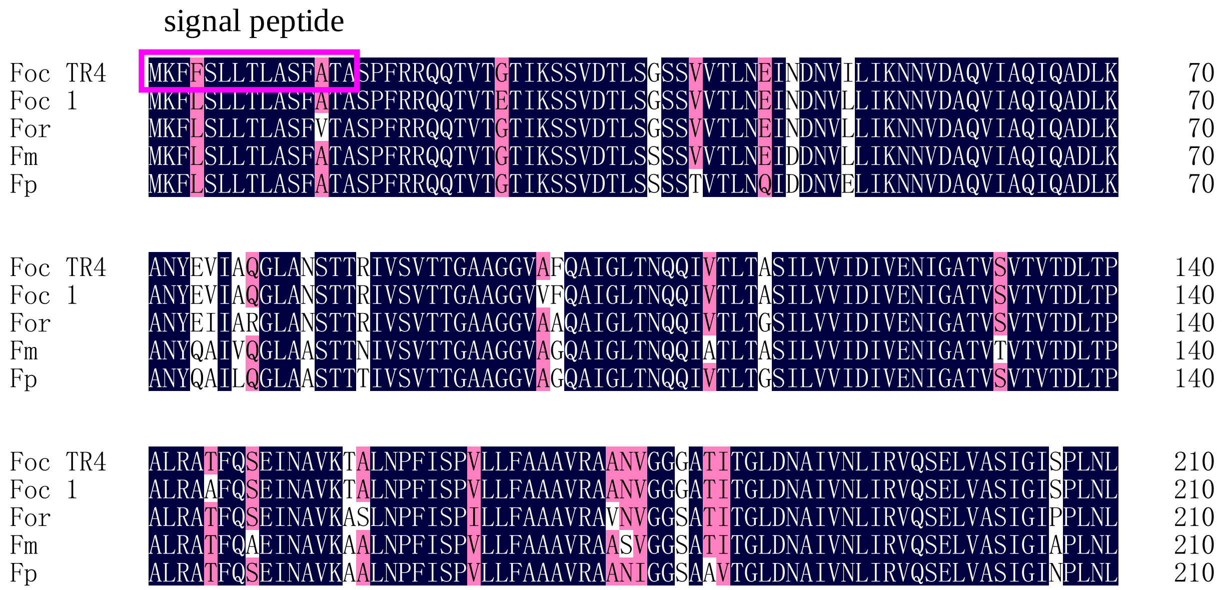

Supplemental Figure S1: Amino acid sequence alignment of FoUpe9 and its homologs in other species, including *F. oxysporum* f. sp. *cubense* race 1 (ENH69994.1; Foc1), *F. oxysporum* f. sp. *rapae* (KAG7418753.1; For), *F. mangiferae* (XP041679195.1; Fm), *F. phyllophilum* (KAF5539974.1; Fp). Identical and conserved amino acid residues are shaded in black.

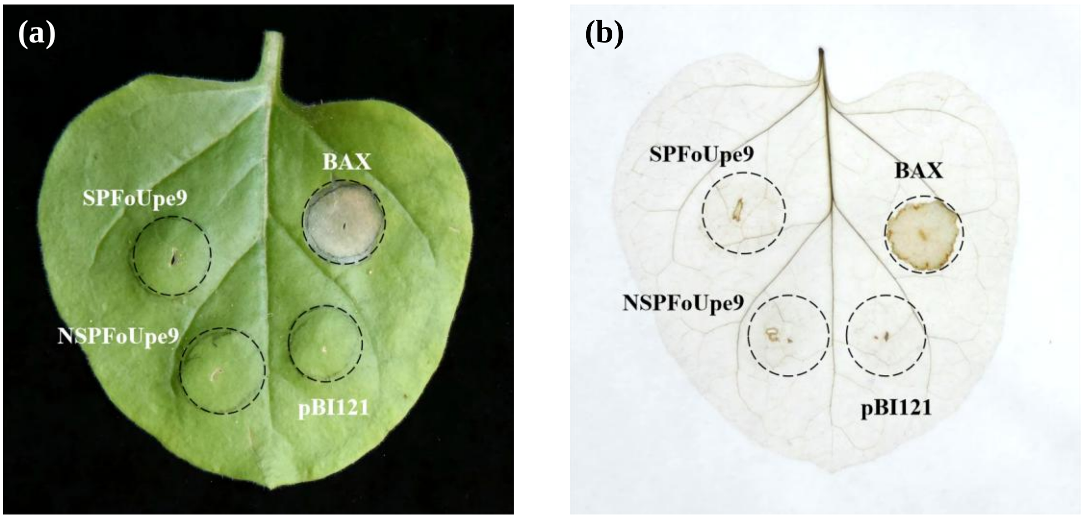

Supplemental Figure S2: FoUpe9 could not induce cell death (a) and ROS accumulation (b) in *N. benthamiana*. *N. benthamiana* leaves were infiltrated with *A. tumefaciens* expressing SPFoUpe9, NSPFoUpe9, BAX (as a positive control), or pBI121-HA empty vector (as a negative control), respectively. The cell death was photographed 3-4 days after infiltration. ROS accumulation was detected by DAB staining.

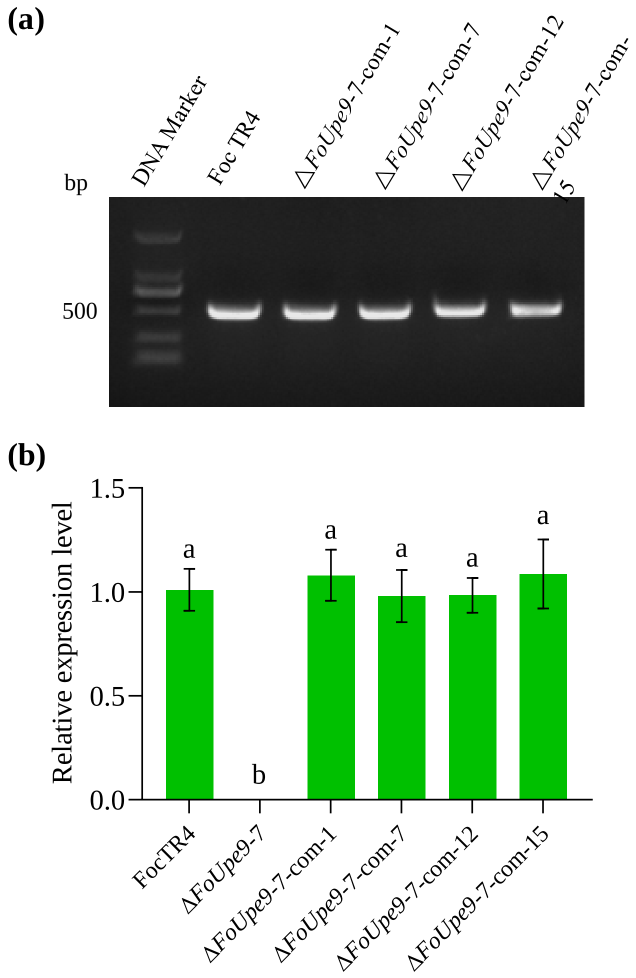

Supplemental Figure S3: PCR (a) and RT-qPCR (b) confirmation of four *FoUpe9* complementation strains. PCR confirmation in (a) using *FoUpe9* as a primer. Values are the means based on three independent experiments, and bars indicate standard deviations. Different letters indicate statistical significance ($p < 0.05$) using Duncan's new multiple-range method.

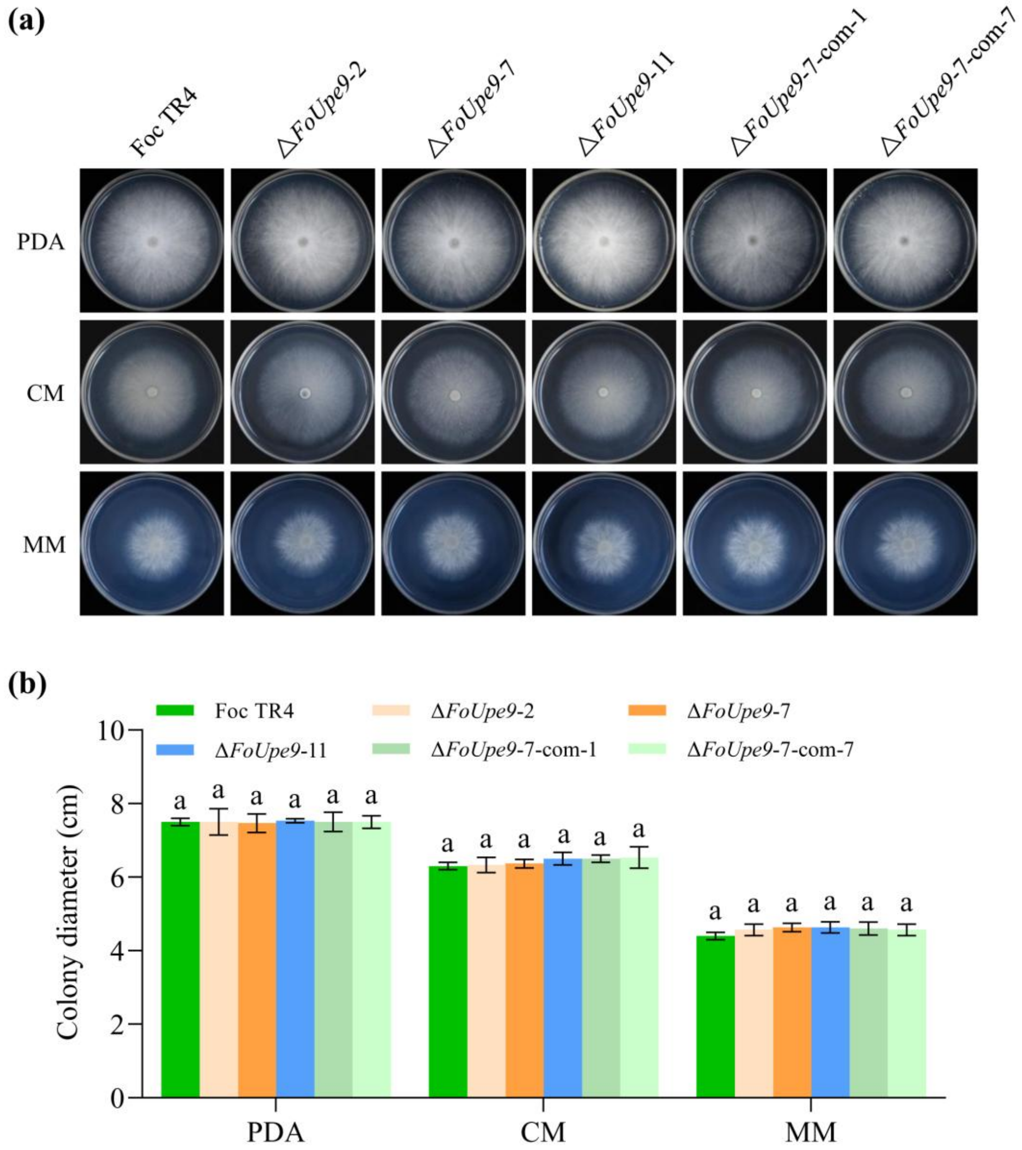

Supplemental Figure S4: FoUpe9 is not essential for the mycelial growth of Foc TR4. (a) Mycelial growth of WT, *FoUpe9* deletion mutants, and *FoUpe9* complementation strains grown on PDA, CM, and MM media at 28 ℃ for 5 days in the dark. (b) Quantification of colony diameters. Values are the means based on three independent experiments, and bars indicate standard deviations. Different letters indicate statistical significance ($p < 0.05$) using Duncan's new multiple-range method.

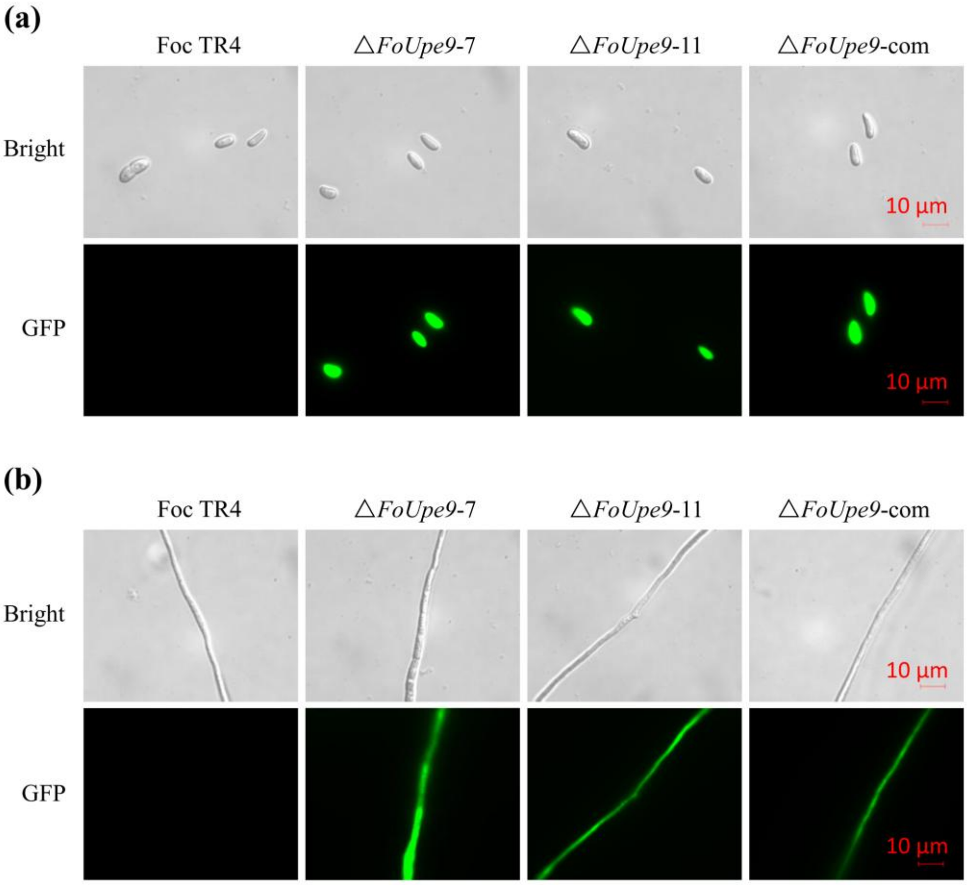

Supplemental Figure S5: FoUpe9 is not essential for the conidiation of Foc TR4. (a) Conidiation of WT, *FoUpe9* deletion mutants, and *FoUpe9* complementation strain cultured in Czapek-Dox liquid media at 120 rpm at 28 ℃ for 3 days. (b) Conidia germination rate was measured after 7 h of incubation in CM media at 120 rpm at 28 ℃. Values are the means based on three independent experiments, and bars indicate standard deviations. Different letters indicate statistical significance ($p < 0.05$) using Duncan's new multiple-range method.

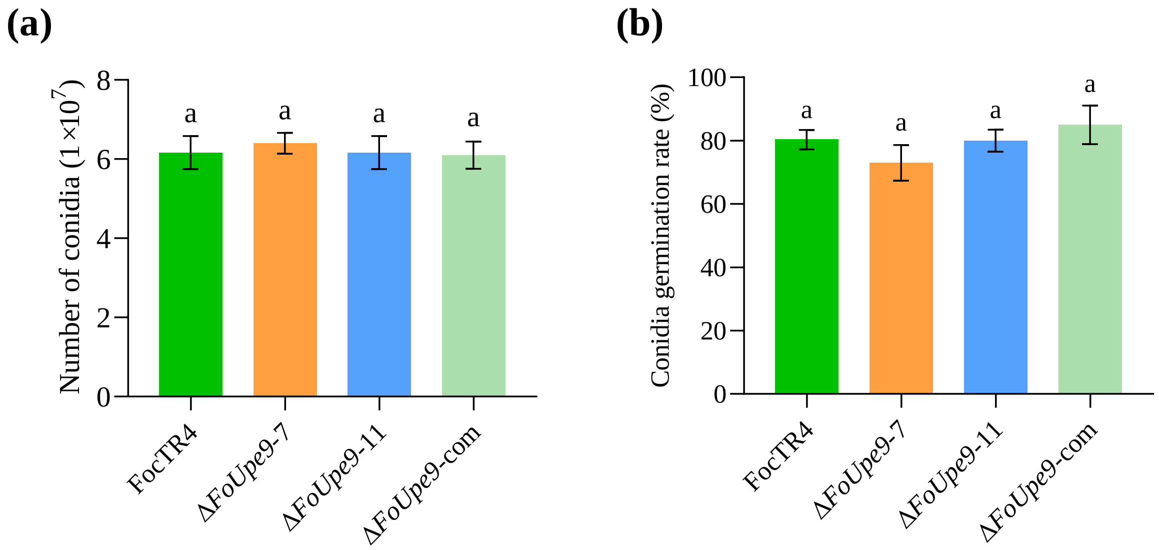

Supplemental Figure S6: Conidial morphology (a) and mycelial morphology (b) of WT, *FoUpe9* deletion mutants, and *FoUpe9* complementation strain were photographed by fluorescence microscope.

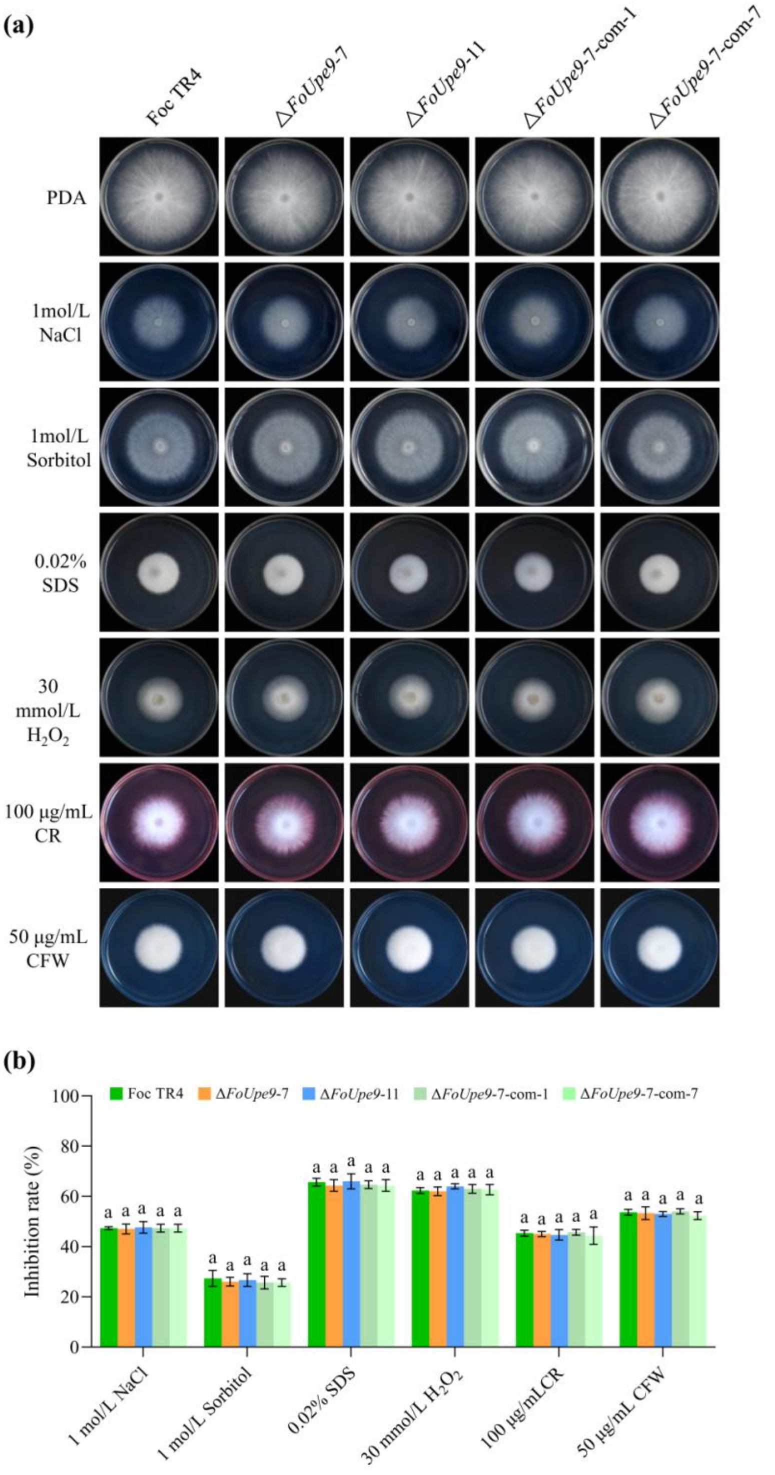

Supplemental Figure S7: FoUpe9 is not sensitive to various stresses. (a) Mycelial growth of WT, *FoUpe9* deletion mutants, and *FoUpe9* complementation strains grown on PDA media amended with 1 mol/L NaCl, 1 mol/L sorbitol, 0.02% w/v sodium dodecyl sulfate (SDS), 30 mmol/L H_2O_2, 100 μg/mL Congo red (CR), and 50 μg/mL Calcofluor White (CFW). All plates were cultured at 28 ℃ for 5 days in the dark. (b) Quantification of colony diameters. Values are the means based on three independent experiments, and bars indicate standard deviations. Different letters indicate statistical significance ($p < 0.05$) using Duncan's new multiple-range method.
